# A Review of High-Throughput Field Phenotyping Systems: Focusing on Ground Robots

**DOI:** 10.34133/2022/9760269

**Published:** 2022-06-16

**Authors:** Rui Xu, Changying Li

**Affiliations:** ^1^Bio-Sensing and Instrumentation Laboratory, College of Engineering, The University of Georgia, Athens, USA; ^2^Phenomics and Plant Robotics Center, The University of Georgia, Athens, USA

## Abstract

Manual assessments of plant phenotypes in the field can be labor-intensive and inefficient. The high-throughput field phenotyping systems and in particular robotic systems play an important role to automate data collection and to measure novel and fine-scale phenotypic traits that were previously unattainable by humans. The main goal of this paper is to review the state-of-the-art of high-throughput field phenotyping systems with a focus on autonomous ground robotic systems. This paper first provides a brief review of nonautonomous ground phenotyping systems including tractors, manually pushed or motorized carts, gantries, and cable-driven systems. Then, a detailed review of autonomous ground phenotyping robots is provided with regard to the robot's main components, including mobile platforms, sensors, manipulators, computing units, and software. It also reviews the navigation algorithms and simulation tools developed for phenotyping robots and the applications of phenotyping robots in measuring plant phenotypic traits and collecting phenotyping datasets. At the end of the review, this paper discusses current major challenges and future research directions.

## 1. Introduction

Plant phenotyping is an emerging science that links genomics with plant ecophysiology and agronomy. Assessments of plant phenotypes in the field can be labor-intensive and inefficient. The emergence of high-throughput field phenotyping (HTFP) is to increase the throughput by leveraging sensing technologies and data processing algorithms. The HTFP systems integrate sensors with mobile platforms to collect data in the field with minimal or no human intervention. Many HTFP systems have been developed so far, including aerial and ground systems. While aerial systems can provide higher efficiency and coverage than ground systems, ground systems usually have a higher payload to carry heavy sensors and equipment and can collect high-resolution data for measuring phenotypic traits at finer levels (e.g., the plant and organ level) and with more viewing angles than aerial systems. In addition, ground platforms typically provide better data quality than aerial systems by controlling the data collection environment (such as light conditions) with well-designed enclosures, and they are less affected by the wind to maintain a straight path to perform more even longitudinal scanning than aerial systems. Early ground HTFP systems were based on tractors and human-operated pushcarts. The recent trend is to use autonomous phenotyping robots to automate data collection.

**Figure 1:**
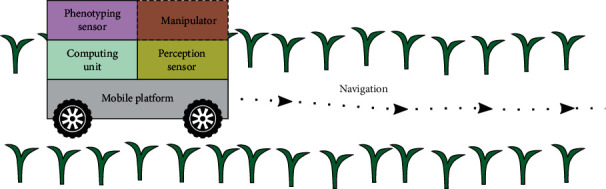
Diagram of a phenotyping robot.

Most agricultural environments are unstructured that can change rapidly in time and space; therefore, a phenotyping robot needs to be intelligent to operate by itself. A robot's intelligence follows a “sense-think-act” cycle where the robot needs to understand its surrounding environment, to make decisions, and to perform certain operations to achieve its goals. For phenotyping robots, the intelligence mainly focuses on navigation in unstructured environments because the phenotyping robot's primary mission is to collect data autonomously in the field. A typical phenotyping robot primarily consists of the mobile platform, sensors including phenotyping sensors for measuring phenotypic traits and perception sensors for navigation, and computing units for data collection and robot navigation (Figure 1). Manipulators, such as robotic arms, are sometimes used in phenotyping robots to measure certain phenotypic traits.

There are a few excellent review papers on robotic technologies for plant high-throughput phenotyping [1, 2], but they either do not solely focus on infield systems or lack detailed review of technical aspects of field robots. Thus, the goal of this paper is to fill the gap and review the existing ground phenotyping robots for HTFP focusing on technical perspectives. First, this paper provides a brief review of the ground phenotyping systems using nonrobotic systems and then a detailed review of ground phenotyping robots with regard to the robot's main components, including mobile platforms, sensors, manipulators, computing units, and software. It then reviewed the navigation algorithms and simulation tools developed for phenotyping robots and the applications of phenotyping robot. At the end, this paper discusses current challenges and presents future research directions for phenotyping robots. This paper is focused on phenotyping robots developed in academia and does not include commercial robots. The reviewed papers were searched using Google Scholar and Web of Science covering literature from the year of 2011 to 2022. We used “high throughput phenotyping”, “robot”, and “robotics” as keywords to search relevant studies. In total, 117 papers including 90 journal papers from 34 different journals and 27 conference papers were selected and reviewed.

## 2. Field-Based Ground Phenotyping Systems

The earliest ground HTFP systems developed were based primarily on tractors because of their wide availability and ease in modification for mounting sensors. However, the soil compaction created by tractors makes them unsuitable for frequent data collection. Therefore, a lightweight pushcart, or motorized cart, was developed to replace tractors. Since tractors and pushcarts still need manual control, the gantry and cable-driven platforms were developed to automate data collection fully. However, gantry and cable-driven platforms are not mobile and can only cover certain fields, limiting the number of experimental plots. Their high construction and maintenance costs also limit their usage.  Table 1 lists the major ground HTFP systems in literature and compares their strengths and limitations.

### 2.1. Tractor-Based Systems

Early tractor-based HTFP systems were modified from high-clearance tractors mounted with multiple sensors to measure plant phenotypic traits. One of the first systems was developed by USDA Maricopa Agricultural Center in Arizona. Their system had eight sets of infrared thermometer and ultrasonic sensors that measure the temperature and height of the canopy of eight rows with a single pass [5]. A Real-time Kinematic Global Navigation Satellite System (RTK-GNSS) was mounted onto the tractor to georeference measurements. Another representative system is BreedVision, which used a tractor with an enclosure to carry multiple sensors [4]. The BreedVision integrated a 3D time-of-flight camera, a light curtain sensor, a laser distance sensor, a hyperspectral camera, and a RGB camera. As imaging technologies advanced, the later developed HTFP platforms mainly used imaging sensors to collect data. For example, the GPhenoVision is a tractor-based multisensor system that integrated a hyperspectral, thermal, and RGB-D camera [9]. The GPhenoVision system has been used to measure morphological traits for cotton [21].

The high payload capacity of the tractor-based HTFP system eases the carrying of heavy equipment, such as the imaging chamber, so that the environmental conditions can be controlled partly for data collection. However, the tractor's heavy weight can create soil compaction with frequent data collection, which could interrupt the crop's growth. Additionally, the data acquisition system can suffer from the tractor's vibrations, which potentially could damage the sensors and affect the data quality if the vibrations are not properly isolated. The field soil conditions (such as muddy soil after rain) could limit the operation of the tractor. Because tractors need to be driven manually, the lack of precise controls for the tractor's speed and trajectory can affect specific sensors, such as the push-broom hyperspectral camera. Furthermore, there is the potential risk for damaging the crops with manual driving, but this is a common issue for manually controlled, ground HTFP systems.

### 2.2. Pushcart and Motorized Cart

As an alternative to the tractor, pushcart can be assembled easily with low-cost materials. It was developed to solve the soil compaction issue of the tractor. Most cart-type systems were made of metal frames with bicycle wheels, which makes them low cost and lightweight [10–15]. The frame structure eases the mounting of sensors. Since the pushcart is activated manually, its position is easier to control than that of tractors such that it can stop at any position and scan the field [14]. However, manual driving makes pushcarts impractical for large fields. The motorized cart uses electronic motors to move the cart, meaning it can be controlled remotely. “Professor” is a platform that uses two DC motors for driving and two DC motors for steering [15]. Its frame is made of aluminum extrusions, and its width and height can be adjusted with an inner frame. It is manually controlled with a remote controller. A self-propelled electric platform was developed recently for wheat phenotyping, and it can carry a person to manually drive it [16]. The major drawback of the pushcart and motorized cart is that they require manual power and manual control, which is inefficient and not practical for large fields. Similar to tractor-based systems, manually controlled pushcart and motorized cart also have the potential risk for damaging the crops with frequent data collection.

### 2.3. Gantry and Cable-Driven System

A gantry is an overhead, bridge-like structure that supports equipment such as a crane. The gantry-based HTFP system uses a gantry to carry sensors and can move linearly on parallel rails. The camera can move along the gantry bridge as well as vertically, making the camera move in XYZ axes. One well-known gantry-based system is the Field Scanalyzer that was developed by LemnaTec [22] and one such system was built at University of Arizona's Maricopa Agricultural Center [17]. The high payload (500) enables the system to carry heavy sensors, such as the chlorophyll fluorescence imager (120). PhÃ©noField is a gantry system managed by the applied research institute ARVALIS in France that was used primarily for wheat breeding [18]. It has a mobile rainout shelter equipped with irrigation booms to control the water stress for desired plots.

Like the gantry-based system, the cable-driven system is a fixed-site system where the sensors were suspended from cables supported by towers at the outside corners of the field. The movements of the sensors were driven by cable winches. Some representative systems include the field phenotyping platform (FIP) at the Swiss Federal Institute of Technology in Zurich [19] and the NU-Spidercam from the University of Nebraska-Lincoln [20].

The advantages of gantry-based and cable-driven platforms are that they do not make physical contact with the soil or plants in addition to being fully autonomous, where the sensors can be precisely controlled at a particular position to scan the crops and can scan the field repeatedly throughout the growing season. The primary disadvantage is the limited field coverage and a fixed experimental site, which can limit their usage in breeding programs. Since the systems usually scan the top view of the canopy, they do not provide information under the canopy without side views. Other disadvantages include high construction and maintenance costs.

## 3. Phenotyping Robot

**Figure 2:**
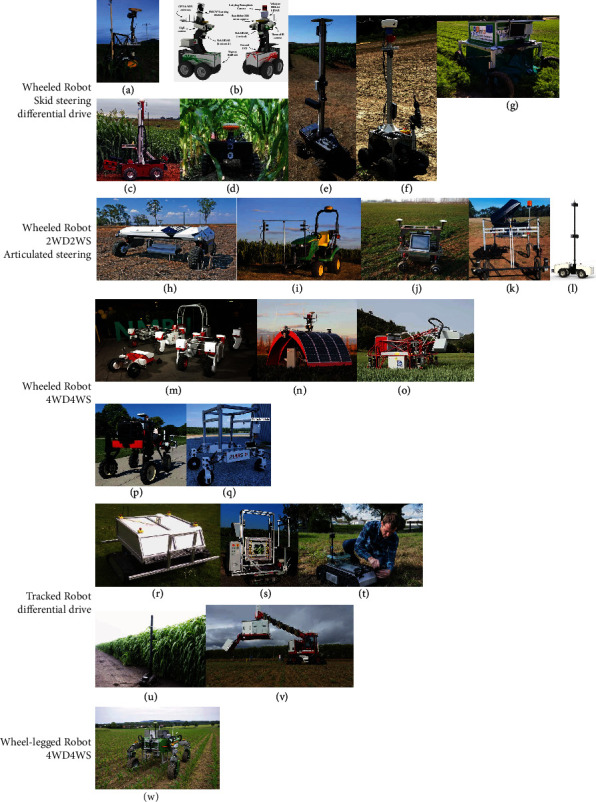
Phenotyping robots. (a) Vinobot [24]. (b) Shrimp [25]. (c) Robotanist [26]. (d) TerraSentia [27]. (e) VinBot [28]. (f) MARIA [29]. (g) RobHortic [30]. (h) AgBotII [31]. (i) Phenobot 1.0 [32]. (j) AgriRover-01 [33]. (k) DairyBioBot [34]. (l) Phenobot 3.0 [35]. (m) Thorvald II [36]. (n) Ladybird [37]. (o) Phenomobile V1 [38]. (p) Flex-Ro [39]. (q) MARS X [40]. (r) Armadillo Scout [41]. (s) PHENObot [42]. (t) A robot based on LT2 [43]. (u) TERRA-MEPP [44]. (v) Phenomobile V2 [45]. (w) BoniRob [46].

The development of agricultural robots has advanced significantly in the past decade to address labor shortages in agricultural. The advantages of automation make the agricultural robot a promising means for managing large farms with minimal human labor and make it an ideal solution for HTFP. Thus, there is a trend to develop phenotyping robots to replace tractors and pushcarts (Figure 2).

### 3.1. Mobile Platform

Existing phenotyping robots can be classified into three categories based on the drive mechanism: wheeled robot, tracked robot, and wheel-legged robot. The wheeled robot uses wheels to drive the robot while the tracked robot uses tracks. These are the two mostly used forms. The legged robot uses articulated legs to provide locomotion, such as a hexapod robot [23]. The wheel-legged robot combines the wheels with articulated legs, so it has more control of locomotion.  Table 2 summarizes some developed phenotyping robots mentioned in the literature.

#### 3.1.1. Wheeled Robot

The wheeled robot is the most common phenotyping robot. Wheeled robots can be classified broadly into two categories: robots with locally restricted mobility (such as skid steering and differential drive robots) and robots with full mobility (such as omnidirectional robots). Skid steering and differential drive robots are the most common robots used for phenotyping because of their simplicity in mechanical structure and motion control [24–28]. Commercial robotic platforms, such as Jackal and Husky from Clearpath, are skid steering robots commonly used for HTFP [24, 72]. Differential drive robots use two drive wheels and passive caster wheels for support [30]. The locomotion of the skid steering and differential drive robot is controlled by the forward/backward and turning speeds of the wheels. It can achieve in-place rotation. Because the turning of skid steering relies on the necessary sliding of the wheel in the lateral direction, skid steering robots have low power efficiency in turning and high wear and tear of tires and can disturb the soil. The difference in rolling resistance or traction in the wheels of a differential drive robot can make the robot turn unexpectedly, making the robot less precise in steering control.

Some wheeled robots use two wheels for steering and two wheels for driving (2WD2WS) [31, 34]. The locomotion is controlled by the forward/backward speed and turning angle, similar to an Ackermann steering, enabling precise steering control. One example is the Phenobot 1.0, which was modified from a small tractor [57]. Its redesigned version, Phenobot 3.0, uses articulated steering [35]. Articulated steered robot has good off-road performance in which the robot was divided into front and rear halves which are connected by a vertical hinge and the steering is controlled by the angle of the two halves. The mechanical complexity of articulated steered robots is increased compared to 2WD2WS robots.

Unlike the above-mentioned robots with restricted locomotion, omnidirectional robots can move in any direction without restrictions, enabling extra maneuverability and terrain adaptation and at the same time increasing the cost and complexity in mechanical structure and motion control. The Thorvald II [36] and Ladybird [37] are two representative four-wheel drive and four-wheel steering (4WD4WS) robots. The modular agricultural robotic system (MARS) is a recently developed phenotyping robotic system featuring a low-cost 3D printable design (MARS mini) and a high-payload high-clearance 4WD4WS robot (MARS X) [40].

#### 3.1.2. Tracked Robot

Tracked robots use tracks to increase the contact area with the ground, and thus, its terrain adaptability is better than the wheeled robot. It can operate on rough terrains and soil conditions (e.g., muddy fields) that wheeled robots are not capable of due to low ground pressure. The kinematics and control of the tracked robot are similar to those of the differential drive wheeled robot. Armadillo and its improved version, Armadillo Scout, are tracked robots featuring a modular design for the track module and a robot computer platform FroboBox running the modular robot architecture, FroboMind, based on ROS [41]. TERRA-MEPP is a tracked robot designed for phenotyping energy sorghum [44]. It uses a tracked platform to carry a vertical, extendable mast (up to 4.88), so the sensor can capture the top view of plants. Phenomobile V2 is a heavy-duty tracked robot that carries a telescopic boom to raise the height of the measurement head mounted on the boom [45]. Commercial tracked robot platforms, such as LT2 from SuperDroid Robots, were used in some studies [43, 65].

#### 3.1.3. Wheel-Legged Robot

Wheel-legged robots combine the advantage of the wheeled and legged robot. It offers the speed as high as the wheeled robot and the high terrain adaptability of the legged robot. Wheel-legged robot can achieve high maneuverability and adjust the robot's dimensions (width and height) to adapt to different field layouts [73]. One well-known wheel-legged robot is the BoniRob, which has four legs with omnidirectional wheels [67]. This robot can adjust its width and height by adjusting the legs' posture and can achieve the same maneuverability as a 4WD4WS robot. BoniRob has a detachable module that reconfigures the robot to perform different tasks by changing the module. The downside of the wheel-legged robot is that its complexity increases the cost of the robot and makes it less robust than a wheeled robot. The increased cost makes it uneconomical since the added benefits of wheel-legged robot is not essential for most phenotyping projects.

### 3.2. Sensors and Manipulators

The primary function of a phenotyping robot is to measure phenotypic traits, so the robot usually carries multiple sensors to capture related information for phenotypic traits. Furthermore, sensors enabling the robot to self-drive and avoid obstacles are necessary. Manipulators are needed when making contact and destructive measurements for certain phenotypic traits, such as the stalk strength of sorghum [26].

#### 3.2.1. Sensors

The sensors used in phenotyping robots include the phenotyping sensors for measuring phenotypic traits and perception sensors for navigation. The phenotyping sensors and the perception sensors can be interchangeable or be independent. The perception sensors are used primarily for localization and path planning. The phenotyping sensors include noncontact sensors, such as imaging sensors, and contact sensors, such as a penetrometer. The most widely used noncontact sensors are the RGB camera, multispectral camera, hyperspectral camera, thermal camera, stereo camera, RGB-D camera, and LiDAR sensor [74, 75]. Most phenotyping robots provide mounting points to carry different sensors according to the targeted phenotypic traits. Some phenotyping robots carry environmental sensors such as soil sensors to measure environmental parameters which are useful metadata for data processing [24, 29, 37].

RGB cameras are the most widely used phenotyping sensor, and RGB images can be used to measure many traits of the crops, such as morphology of the plants [24] and plant organ count [25, 49, 76]. The image quality can be affected by the natural illumination in the field so a light chamber can be used to control the lighting [76]. Artificial lighting can be used when collecting data at night, which can effectively remove the background crops in the image [66]. Strobe lights can be used in the daytime to enhance the foreground [48]. Stereo cameras and RGB-D cameras can provide depth measurements other than RGB images so they can be used to measure 3D structure of the plants. With the depth information, the 3D morphology of the plants can be measured such as canopy size [21] and plant architecture [57, 77]. The depth information can assist the detection and counting of fruits for horticulture crops and estimate the size of the fruits [78]. Similar to RGB cameras, the depth measurement can be greatly affected by the illumination conditions, especially for the RGB-D cameras that use structured light [79]. Therefore, properly controlling the lighting condition in the field is important to improve the measurement accuracy.

Multispectral, hyperspectral, and thermal cameras can provide more spectral information about the crops than an RGB camera. Multispectral and hyperspectral are typically used to measure phenotypic traits that are related to the spectral reflectance of the plants. For example, vegetation indices derived from certain spectral bands like NDVI are related to the physiological activities of the plants, which can be used to detect plant disease [30], abiotic stresses [80], and fruit maturity [81]. Thermal camera is typically used to measure the temperature of the plants, which is correlated with the water status of the plants [80]. Similar to other imaging sensors, multispectral and hyperspectral cameras are affected by the sunlight in the field, which requires in-field calibration to get correct spectral reflectance of the plants. Thermal imaging is less sensitive to sunlight but more easily affected by the atmospheric condition so the environmental conditions should be recorded to calibrate the thermal image.

LiDAR sensors measure the distance to the target based on the time-of-flight principle using an active laser pulse. Thus, it is not limited by the lighting conditions and covers a larger sensing range than the stereo and RGB-D camera. Each laser scan can generate the shape profile of the plants of one layer from 2D LiDAR or multiple layers from 3D LiDAR. Registration of the laser scans using their position and posing generates a 3D point cloud of the plants, which can be used to measure the morphological traits of the plants [34]. Therefore, accurate localization of the robot is important for the registration of the laser scans.

#### 3.2.2. Manipulators

Manipulators, primarily robotic arms, are used commonly in agricultural robots, such as weeding and harvesting robots. However, manipulators are not very common in phenotyping because most phenotypic traits can be measured remotely. Manipulators are useful for phenotyping robots when the phenotypic traits need to be measured in contact or at a specific location (e.g., certain leaf). For example, the Robotanist robot uses a three degree-of-freedom robotic arm to measure the stalk strength of sorghum [26]. Sensors mounted on the robotic arm can be used to change sensing position/pose actively, such as sensing individual plants from multiple viewing angles [24, 82, 83]. Other applications include collecting biological samples [84], leaf probing [85], soil sampling [29, 68], digging plants for root phenotyping, and fruit mapping [86].

### 3.3. Computing Unit and Software

The computing unit in phenotyping robot has two main tasks: performing autonomous navigation and collecting the phenotyping data. They are sometimes independent of each other. Single-board computers and embedded systems are used commonly in robotic systems because of their small size, low power consumption, and lightweight. However, their computing resources are usually limited. The selection of a computing unit should consider power consumption, computing performance, size, weight, interfaces, and supported operating system.

Although a single computing unit can be used for both autonomous navigation and data collection, a common design is to use a dedicated computing unit for each task. This design brings two benefits. First, appropriate computing units can be selected based on the computing resources required by each task. For example, some embedded systems that are dedicated to autonomous vehicles such as Pixhawk [87] can be used for autonomous navigation [34]. For collecting phenotyping data, a computing unit with more computing resources (e.g., PC or industrial computer) may be needed to handle the large data volume from imaging sensors. Second, it can make an independent system for data collection so it can be deployed on different robotic platforms. It is also easier to add/replace phenotyping sensors. The drawback of using several computing units is the higher communication overhead and hardware costs than using a single computing unit.

The Robot Operating System (ROS) is a widely used middleware framework for developing robotic software because it provides an integrated environment that can greatly accelerate software development [88]. ROS has become an industrial standard for robotics and supports a wide range of hardware and algorithms commonly used in robotics, but with constraints such as not supporting real-time control. The ROS 2, a newer version of ROS, was developed to support real-time control, microcontroller, and multiple robots and platforms [89]. FroboMind is a software architecture built upon ROS and designed for agricultural robots [90]. LabVIEW was used by some robots for control and data collection [39, 43]. Other robot software architectures can be found in [90].

### 3.4. Navigation

Navigation is an essential component of automation in robotics and includes three fundamental problems: localization, path planning, and map building. A typical agricultural environment includes many crop rows in straight lines, and the robot needs to travel along the crop rows. Therefore, a phenotyping robot's primary navigation objective is to follow the crop row and switch between rows. GNSS, vision sensors, and LiDAR sensors are commonly used for localization and path planning. GNSS and IMU can be used to obtain the global position and posing. Vision sensors and LiDAR sensors can be used for localization and obstacle detection using the simultaneous localization and mapping (SLAM) algorithms [91]. This paper focuses on the navigation algorithms based on these sensors in the agricultural environment. Other navigation methods such as magnetic-based navigation that are not commonly used for agricultural robots were not reviewed.

#### 3.4.1. GNSS-Based Navigation

As a global positioning technology, GNSS has been used widely to localize robots in field applications. GNSS-based guidance systems have been developed for agricultural machinery and robots [92]. The RTK-GNSS can provide positioning accuracy up to a centimeter but is not always adequate for localization when used as a single positioning sensor. The positioning accuracy of GNSS can be affected by the obstruction of line-of-sight to satellites, multipath issues, and interference from other radio frequency (RF) sources. In addition, GNSS does not provide accurate heading measurement. Therefore, it is typically used with other sensors, such as the IMU and wheel encoder, to improve the localization accuracy.

The typical application of GNSS-based navigation is to make the robot follow preset paths using path-following algorithms, such as pure pursuit controller and its variants [93]. The path-following algorithm can be designed using conventional control theories, which require the robot's kinematic model [64]. Deep reinforcement learning can also be used for following paths, which does not require the robot's kinematic model, and can learn the kinematics implicitly through training [94].

In agricultural environments such as orchards, the GNSS can be unreliable because the robot frequently could move under a tree canopy blocking the satellite's signals to the GNSS receiver. The GNSS-based navigation is not suitable for dynamic environments with unexpected changes or events in the environment. In those cases, vision-based and LiDAR-based navigation algorithms can be used.

#### 3.4.2. Vision-Based Navigation

Vision-based navigation keeps the robot following crop rows using machine vision. RGB cameras typically are used to detect crop rows and calculate the robot's orientation relative to the crop row [95]. Stereo vision can provide depth information, which can help detect crop rows with different illumination conditions and weed pressure than a single camera [96]. Besides the traditional machine vision techniques, the deep learning methods can obtain directly the crop row's orientation from raw images [97].

Vision-based navigation relies on the image feature of the crop rows and can suffer from illumination changes and lack of texture [96]. Typically, it is used with GNSS guidance to improve the robustness, for example, fusing the vision guidance and GNSS guidance results or using the vision guidance for row following and switching to GNSS guidance when the robot shifts between rows.

#### 3.4.3. LiDAR-Based Navigation

LiDAR can measure the distance between objects. Like vision-based navigation, LiDAR-based navigation relies on landmarks that can differentiate crop rows, such as the plant, trunk, and poles in a polytunnel [54, 60, 98]. The crop row measured by LiDAR sensors is represented as points along with some noise. Because the LiDAR sensor is subject to noise, it is difficult to detect crop rows from noisy points. A standard method is to detect the crop row using line detection algorithms, such as Hough transform and random sample consensus (RANSAC) [54, 60]. Another method is to model the LiDAR measurements and noise using a particle filter and estimate the robot's heading and lateral deviation relative to the crop row [99, 100].

Using the LiDAR sensor alone can make it challenging to understand the surrounding environment because of the coarse data. The vision sensor can be used to provide complementary information to exclude the LiDAR points of no interest from data processing. For example, image features were used to separate the LiDAR points of the trunk from other objects in vineyards so that crop rows could be detected correctly [101]. The LiDAR sensor also can be used for obstacle avoidance, but can falsely detect grass, weed, and plant leaves as obstacles, so using vision sensors can help identify real obstacles.

### 3.5. Simulation

**Figure 3:**
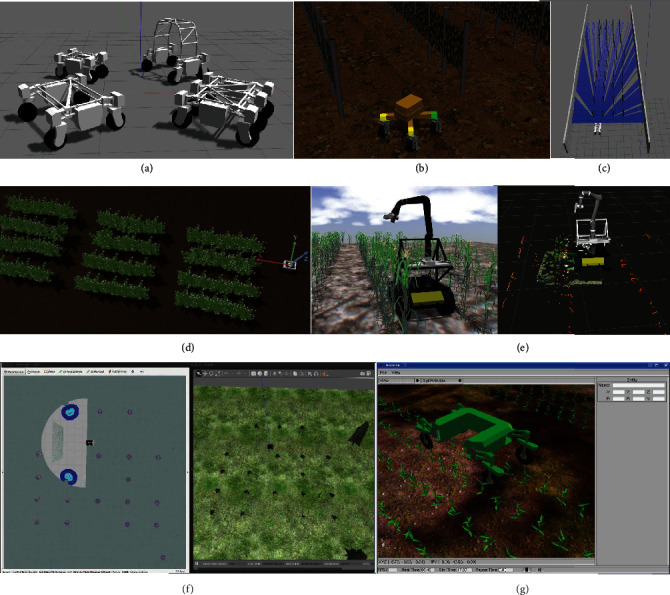
Simulation of various phenotyping robots. (a) Thorvald II robots in Gazebo simulator [102]. (b) Simulation of an omnidirectional mobile robot in a typical vineyard in Gazebo simulator [103]. (c) Simulation of a row-following robot in polytunnels [60]. (d) Simulation of LiDAR-based navigation in row crop using MARIA [54]. (e) Simulation of Vinobot in Gazebo simulator and its visualization in Rviz [51]. (f) Simulation of agriculture field using AgROS [110]. (g) Simulation of BoniRob in Gazebo simulator [46].

Simulation of the robotic system and its operating environment can accelerate the development of robotic systems through quick and efficient tests and validation of the robot's design without physically building the robots (Figure 3) [46, 54, 102, 103]. Simulation is also useful for developing and testing control algorithms, navigation algorithms, and data processing algorithms [60, 94, 103, 104]. It is easy to create repeatable testing conditions in simulations for the robot, a process that can be difficult in a real environment.

There are many simulation software/platforms, and popular ones include Gazebo [105], Webots [106], and V-REP (now called CoppeliaSim) [107]. All three simulation platforms can provide a complete simulation environment to model and program a wide range of mobile robots and sensors. Gazebo is one of the most popular multirobot simulators which support a wide range of sensors and objects. It is open-source and is compatible with ROS and thus is used by many phenotyping robots for simulation [46, 51, 54, 102, 103]. However, Gazebo currently only supports Linux systems and lacks a good user interface. Webots and V-REP are cross-platform software, support multiple programming languages, and can be interfaced with third-party applications. Webots and V-REP were initially developed by industrial companies and are free to use now. A complete review of the simulation platforms can be found in [108]. Some simulators and frameworks customized for agricultural robotics and farm machinery have been designed based on professional simulation platforms, such as the Agricultural Architecture (Agriture) [109] and AgROS [110].

## 4. Applications of Phenotyping Robot

The primary mission of a phenotyping robot is to measure phenotypic traits of plants. The data collected by the phenotyping robots can be used for various purposes. We grouped the applications into three categories based on the phenotypic traits and usage of the traits. The three categories are crop organ identification and counting, crop detection and classification, and crop growth monitoring, as summarized in Table 3.

### 4.1. Crop Organ Identification and Counting

The high-resolution data collected by the ground phenotyping robots can be used to detect plant traits at the organ level, which cannot be achieved by aerial systems. RGB images can be used to detect the plant organs, such as crop leaf and fruit, using machine learning methods. For example, a customized tracked robot was developed to collect RGB images of kiwifruits and an image processing algorithm using traditional machine learning methods was designed to count the fruits [111]. Deep learning methods can be used to detect plant organs by designing and training appropriate neural networks [112]. A customized neural network model was designed to detect and localize crop leaves using the RGB images collected by BoniRob [113]. Mango fruits were detected and counted from RGB images collected by the Shrimp robot using a Faster R-CNN model [25].

### 4.2. Crop Detection and Classification

Detection of crops and weeds can be used for many applications, such as weed control and plant count. RGB images can be used to detect the plants or weeds using deep learning methods. For example, RGB images from TerraSentia can be used to detect and count the corn stand using Faster R-CNN [27, 114]. When the corn plants grow tall, Robotanist can run between crop rows and count the plants by detecting the corn stalk using Faster R-CNN [50]. The width of the corn stalk can be measured from the stereo images. Plants can also be detected using LiDAR sensor by detecting the ground plane and separating the plants using clustering algorithms [115]. Machine learning-based methods for crop and weed detection require large training dataset, which can be collected by phenotyping robots. An image dataset was collected using BoniRob for weed detection in a carrot field [69], and the weeds were detected using Random Forest [116]. BoniRob also was used to collect datasets containing georeferenced multispectral images, and RGB-D images and LiDAR data were collected for plant classification, localization, and mapping in a sugar beet field [70].

### 4.3. Crop Growth Monitoring

The growth conditions of the crops can be reflected by many morphological traits. The 3D model of the plants can be obtained from RGB images using Structure from Motion [24], depth images from stereo or RGB-D cameras [32, 55], or LiDAR sensors [34, 48]. Plant height, width, stem diameter, plant volume, and surface area can be estimated from the 3D model [32, 57]. The corn stalk diameter was estimated using a RGB-D camera, where the RGB image was used to detect the corn stalk and the depth image was used to measure the stalk diameter [55]. The plant volume of perennial ryegrass was measured using a LiDAR sensor on DairyBioBot, which was correlated with the biomass [34]. The canopy volume of almond trees were measured using a LiDAR sensor on the Shrimp robot, which was shown to be correlated with the yield [48]. The flower and fruit density of the almond tree was also measured from RGB images.

## 5. Challenges and Future Perspectives

### 5.1. Challenges

Despite recent advances in sensors and robotics, designing a phenotyping robot that can work in unstructured and dynamically changing agricultural environments can be challenging. There remain several major challenges. First, some phenotyping robots have been designed for specific crops and field layouts, which limits their use in other crops and field layouts. For example, robots designed for vineyards, such as PHENObot, may not be suitable for row crops because the robots' dimensions cannot fit within the row spacing [42]. The changes in height and size of the plants due to growth also limit the usage of the robot throughout the growing season. For example, it can be difficult to run a robot between crop rows without damaging the plants when the canopy grows into each other. The robot's design (e.g., the dimension of the robot) is constrained by agronomic practices such as row spacing and the dimension of the crops, which usually vary crop by crop, making it challenging to design a robot to work properly under the constraints without sacrificing the functionality. Second, the costs of phenotyping robots are prohibitively high in most cases [1]. The mobile platform itself may cost tens of thousands of dollars, and the total cost of a phenotyping robot is even higher with perception and phenotyping sensors [117]. Although some low-cost robots, such as the TerraSentia, have been developed, their use has been limited because of the low payload and small size of these robots. Third, the data collection efficiency of phenotyping robots remains too low for large fields with tens of thousands of plots in practice. For example, a single robot would take at least 1.7 hours to scan 1000 plots of 3 m length at a travel speed of [per-mode = repeated-symbol] 0.5. The lengthy scanning time can make time-sensitive traits (e.g., canopy temperature) unreliable across plots. Fourth, navigation in cluttered environments is challenging, especially in GNSS-denied areas such as under subcanopy. A complex navigation algorithm using vision or LiDAR is needed for those environments [118]. Fifth, data processing and phenotypic trait extraction are mainly done offline in most cases, which is not usable for real-time decision-making and online control. More robust and efficient perception and control methods are needed. Six, regulation and robot safety should be taken into consideration when designing and operating the robot, which can potentially increase the operation cost of the robot and limit its usages in some countries/areas [119, 120].

### 5.2. Future Perspectives

To address the above-mentioned challenges and further advance automated phenotyping, there are several future research directions for phenotyping robots. First, it is important to develop reconfigurable robots with a modular design to adapt to different cropping systems in terms of plant height, row spacing, and field layout. A few researchers and companies have developed multipurpose modular robotic platforms such as BoniRob, Thorvald II, and MARS [40]. The modular design in both hardware modules and software modules enables the robots to be flexible in operating in various environments, for instance, greenhouses, polytunnels, and open fields. In addition to be flexible, modularity also brings several other benefits including (1) reduced total cost by reusing the modules to perform phenotyping tasks for different crops and (2) easier and inexpensive maintenance by replacing and repairing only the failed modules without changing the whole robot.

Second, innovative mechanical designs of the mobile platform can be explored to improve the data quality in fields with complex terrains. One promising research direction is legged robot. Not many legged robots have been developed for agricultural purposes due to the complexity of controlling the robot's locomotion and its low efficiency working on large farms [121]. The recent advances in robotic technologies and the commercial success of legged robots, such as the Spot from Boston Dynamics, demonstrated its potential for HTFP [122]. Low-cost open-source quadruped robots from academic institutions, such as the Mini Cheetah, also open the possibility to customize the legged robots for HTFP [123].

Third, to address the low-throughput issue for a single mobile robot, one solution is to deploy a team of heterogeneous autonomous mobile robots (i.e., robot swarms) to work collectively and cooperatively to cover a large field. The heterogeneous robots may possess different sensing capabilities (e.g., multispectral imagery for plant stress detection and LiDAR for plant growth monitoring), internal characteristics (e.g., payload, speed, and robot dynamics), and available resources (e.g., remaining battery power). Researchers have investigated this problem with the distributed coverage control approach that models the field as a weighted directed graph and uses partitioning algorithm to assign the tasks to each agent optimally [124]. Coordination between UGV and UAV has been demonstrated to achieve the best efficiency by combining the benefits of ground and aerial systems [71]. For example, UAV can quickly scan the field to find the areas of interest that need further scans for UGV, reducing the overall data collection time for UGV by focusing on the areas that require high-resolution data.

Fourth, we envision that robust low-cost global positioning method for navigation in complex and GNSS-denied environments will replace expensive Real-Time Kinematic (RTK) GNSS-based navigation. One promising solution is to fuse multiple consumer-grade low-cost sensors (such as low-cost GPS and stereo camera for visual odometry) with additional constraints such as digital elevation model provided by UAV and leverage the 6D pose graph optimization method to achieve accurate and reliable global positioning for mobile robots [118]. The benefits of this approach are multifaceted: it is low cost and more robust against issues such as multipath interference, and most importantly, it can provide the full 6D pose (translation and rotation) that conventional RTK GNSS cannot provide. It is expected that more research advancements in this direction will occur in the coming years.

Fifth, deep learning is expected to have a significant impact on phenotyping robots in robot perception and control. In terms of robot perception, one type of deep learning model called convolutional neural networks (CNNs) has consistently outperformed traditional machine learning techniques in important computer vision tasks, such as image classification/regression, object detection, and semantic/instance segmentation [112]. CNNs are expected to be deployed on the robot through edge computing for real-time inference to help robot understand the scene and to extract phenotypic traits. In terms of robot control, one important AI technique called deep reinforcement learning is expected to play an increasingly important role in path planning and trajectory following [125].

## Figures and Tables

**Table 1:** Summary of the ground HTFP systems.

System	Sensors	Crop	Phenotypical traits	Advantages	Disadvantages
Tractor-based system				High payload Wide availability Easy to be modified for data collection	Large vibration Heavyweight can create soil compaction Data collection is limited by the soil conditions Manual drive could damage the crops Difficult to control its speed and trajectory
[3]	RGB camera LiDAR Spectrometer	Wheat Cotton	Canopy height NDVI NDRE Canopy temperature Green pixel fraction Fraction of intercepted radiation Chlorophyll content Plant and ear density		
BreedVision [4]	3D camera Light curtain Laser distance sensor Hyperspectral camera RGB camera	Triticale	Plant height Canopy reflectance		
[5]	Infrared temperature sensor Ultrasonic sensor Spectral reflectance sensor	Cotton	Canopy height Canopy temperature NDVI		
[6]	Ultrasonic sensor Spectral reflectance sensor RGB camera Infrared thermometer	Cotton	Plant height Ground cover fraction NDVI Canopy temperature		
Phenoliner [7]	RGB camera NIR camera Thermal camera Hyperspectral camera	Grape	Plant count		
GPhenoVision [8]	RGBD camera Thermal camera Hyperspectral camera	Cotton	Plant height Projected leaf area Canopy volume Canopy temperature In-row width Cross-row width		
ProTractor [9]	RGB camera	Brassica	Seedling count		

Pushcart				Light weight Low cost Easy to control the speed and position	Not practical for large field Manual control could damage the crops
[10]	Ultrasonic sensor NDVI sensor Infrared thermometer Spectrometer RGB camera	Soybean Wheat	Canopy height NDVI NDRE Canopy temperature Green pixel fraction		
Phenocart [11]	Spectral reflectance sensor RGB camera Infrared thermometer	Wheat	Canopy temperature NDVI		
Proximal sensing cart [12]	Ultrasonic sensor Infrared thermometer Spectral reflectance sensor RGB camera	Cotton	Canopy height Canopy temperature NDVI Canopy cover Crop water stress index Leaf area index		
Phenocart [13]	RGB camera NIR camera	Wheat	Biomass NDVI		
[14]	Hyperspectral camera	Tobacco			
Motorized pushcart					
Professor [15]	Not specified	Wheat Maize	Not specified		
[16]	LiDAR sensor Spectral reflectance sensor	Wheat	NDVI Photochemical reflectance index Canopy height		

Gantry system				Fully autonomous No physical contact with the soil or plants Field scanning can be precisely controlled No damaging to the plants	Fixed experimental site Only the top view of the canopy can be scanned High construction and maintenance cost
Field Scanalyzer [17]	Thermal camera Chlorophyll fluorescence imager 3D laser scanner RGB camera Hyperspectral camera	Wheat	Plant morphology Canopy temperature Spectral indices		
PhénoField [18]	LiDAR sensor Spectroradiometer RGB camera	Wheat	Green cover fractions Green area index Average leaf angle Meris terrestrial chlorophyll index Plant height		
Cable system					
Field phenotyping platform [19]	RGB camera NIR camera Laser scanner Thermal camera Ultrasonic sensor Spectrometer Multispectral camera	Wheat	Enhanced NDVI Canopy cover Canopy height Canopy temperature Canopy spectral reflectance		
NU-Spidercam [20]	Multispectral camera Thermal camera Spectrometer 3D LiDAR	Maize Soybean	Canopy cover NDVI Canopy temperature Canopy height Canopy reflectance		

**Table 2:** Summary of phenotyping robots categorized by drive mechanism. 4WD4WS: four-wheel driving and four-wheel steering; 2WD2WS: two-wheel driving and two-wheel steering.

Robot	Phenotyping sensor	Perception sensor	Crop	Advantages	Disadvantages	Applications
Wheeled robot (skid steering)						
VinBot [28]	RGB-D camera 2D LiDAR	RTK-GNSSIMU	Grape	Simple mechanical structure Simple motion control Low cost	Low power efficiency in turning High wear and tear of the tires Can cause damage to the soil	Yield estimation [47]
Shrimp [25]	3D LiDAR RGB camera Hyperspectral camera	RTK-GNSSIMU	Horticultural crop			Mango fruit detection [25] Almond mapping of flower, fruits, and yield [48] Almond fruit detection [49]
Robotanist [26]	Stereo camera RGB camera	RTK-GNSSIMU2D LiDAR Stereo camera	Sorghum			Corn stalk count and width estimation [50]
Vinobot [24]	Trinocular camera	GNSS2D LiDAR	Maize Sorghum			Simulation of Vinobot [51]
TerraSentia [27]	Multispectral camera2D LiDAR Hyperspectral camera RGB camera RGB-D camera	GNSS Gyroscope	Maize			Corn stem width estimation [52] Under canopy navigation [53]
MARIA [29]	2D LiDAR RGB-D camera	RTK-GNSSIMU	Not specified			Simulation of an agricultural robot [54]
[55]	RGB-D camera	RTK-GNSS2D LiDAR	Maize			Corn stalk diameter estimation [55]

Wheeled robot (differential drive)						
RobHortic [30]	Thermal camera Multispectral camera RGB camera NIR camera Hyperspectral camera	RTK-GNSS	Horticultural crop	Simple mechanical structure Simple motion control Low cost	Less precise in steering control	Carrot disease detection [30]

Wheeled robot (2WD2WS and Ackerman steering)						
AgBotII [31]	RGB camera	RTK‐GNSS	Row crop	Precise steering control	Need coordination between drive wheel and steering wheel	Weed detection [56]
Phenobot 1.0 [32]	Stereo camera	RTK‐GNSS	Sorghum			Sorghum plant height and stalk diameter estimation [32] Sorghum plant architecture [57]
AgriRover-01 [33]	3D LiDAR	RTK‐GNSS	Corn			Plant height and row spacing estimation [58]
DairyBioBot [34]	2D LiDAR NDVI sensor	RTK‐GNSS	Ryegrass			Ryegrass biomass estimation [34]

Wheeled robot (articulated steering)						
Phenobot 3.0 [2, 35]	Stereo camera	RTK-GNSS	Sorghum	Good mobility for rough terrain Precise steering control	Increased complexity in mechanical structure	

Wheeled robot (4WD4WS)						
Thorvald II [36]	Application dependent	GPSIMU2D LiDAR	Row crop Horticulture crop	High maneuverability Good terrain adaptation	Complex mechanical structure Complex motion control High cost	Development of a strawberry harvesting robot [59] Development of row-following algorithm in polytunnels [60]
Ladybird [37]	RGB camera2D LiDAR Hyperspectral camera Stereo camera Thermal camera	2D LiDAR RTK-GNSSIMU	Row crop			Weed detection [61] Dataset collection [62]
Phenomobile V1 [38]	2D LiDAR RGB camera Multispectral camera	RTK-GNSS	Row crop Wheat Mixed crop			Estimation of plant height from LiDAR measurement [38] Estimation of wheat green area index from LiDAR measurement [63]
AgRover [64]	Not specified	RTK-GNSS	Row crop			
Flex-Ro [39]	RGB camera Ultrasonic sensor Infrared thermometer Spectrometer	GNSS Obstacle detector	Row crop			
MARS X [40]	Application dependent	RTK-GNSS	Row crop			

Tracked (differential drive)						
Armadillo Scout [41]	Application dependent	GNSS2D LiDAR	Not specified	Good mobility for rough terrain Low ground pressure	Complex mechanical structure Low power efficiency in turning Can damage the soil	
PHENObot [42]	RGB camera	RTK-GNSS	Grape			Grape bunch and berry detection [66]
[65]	RGB camera	RTK-GNSS	Soybean			
[43]	RGB camera					
TERRA-MEPP [44]	Stereo camera Depth camera RGB camera	RTK-GNSS Wheel encoder Gyroscope	Sorghum			
Phenomobile V2 [45]	2D LiDAR RGB camera Multispectral camera	RTK-GNSSIMU	Wheat Cotton Sunflower Mixed crop			

Wheel-legged (4WD4WS)						
BoniRob [67]	Application dependent	RTK-GNSS Inertial sensor3D LiDAR	Row crop	High maneuverability Good terrain adaptation Physical dimension can be changed	Complex mechanical structure Complex motion control High cost	Soil compaction and moisture measurement [68] Weed image dataset collection [69] Image dataset collection [70] Ground and aerial robot collaboration [71]

**Table 3:** Applications of phenotyping robots.

Crop	Key issues	Robot	Phenotyping sensor	Data processing method	Reference
Organ identification and counting					
Almond	Almond fruit detection	Shrimp [25]	RGB camera	Use Faster R-CNN to detect fruit in the color image	[49]
Not mentioned	Plant detection and leaf count	BoniRob [67]	RGB camera	A customized single-stage object detection network based on FCN	[113]
Kiwifruit	Kiwifruit detection	Customized tracked robot	RGB camera	Image features were extracted and classified using machine learning	[111]
Mango	Mango fruit detection, localization, and yield prediction	Shrimp [25]	RGB camera LiDAR sensor	Use FR-CNN for detecting fruits in the color image Use LiDAR point cloud and a hidden semi-Markov model to separate individual tree Use epipolar geometry to track fruits and triangulation for fruit localization	[25]

Crop detection and classification					
Corn	Corn plant detection and mapping	Volksbot RT-3	LiDAR sensor	Detect a plan as ground Cluster the nonground points into individual plants	[115]
Corn	Corn stalk count and stalk width estimation	Robotanist [26]	Stereo camera	Use Faster R-CNN to detect stalk and FCN to get stalk mask Use stalk mask to estimate stalk width	[50]
Corn	Corn stand count	TerraSentia [27]	RGB camera	Use Faster R-CNN to detect corn stand	[27, 114]
Carrot	Weed detection	BoniRob [67]	Multispectral camera	Classification of weed and plant is achieved using the Random Forest classifier	[69, 116]
Sugar beet	Dataset collection for plant classification, localization, and mapping	BoniRob [67]	Multispectral camera RGB-D camera LiDAR sensor		[70]

Crop growth monitoring					
Sorghum	Sorghum height and stem diameter estimation	Phenobot 1.0 [32]	Stereo camera	Reconstruct dense point cloud from stereo image Plant height and stem diameter were extracted from the dense point cloud	[32]
Sorghum	Sorghum height, width, stem diameter, plant volume, and surface area estimation	Phenobot 1.0 [32]	Stereo camera	Use convex hull to estimate plant volume and surface area	[57]
Corn	Corn stalk diameter estimation	Customized skid steering robot	RGB-D camera	Use YOLO V4 to detect corn stalk	[55]
Corn	Plant height, leaf area index estimation	Vinobot [24]	RGB camera	3D point cloud of the plant was constructed using structure from motion Plant height and leaf area index were calculated from the point cloud	[24]
Almond	Mapping canopy volume, flowers, fruit, and yield estimation	Shrimp [25]	RGB camera LiDAR sensor	Use color image to detect flower and fruits Use canopy volume from LiDAR point cloud to estimate yield	[48]
Ryegrass	Ryegrass biomass yield estimation	DairyBioBot [34]	LiDAR sensor	Estimate the plant volume from LiDAR point cloud and correlate with the yield	[34]

## Data Availability

This review paper does not contain research data to be shared.
